# Molecular and Pathophysiological Links between Metabolic Disorders and Inflammatory Bowel Diseases

**DOI:** 10.3390/ijms22179139

**Published:** 2021-08-24

**Authors:** Chang-Kee Hyun

**Affiliations:** School of Life Science, Handong Global University, Pohang 37554, Gyungbuk, Korea; ckhyun@handong.edu; Tel.: +82-54-260-1361

**Keywords:** obesity, type 2 diabetes, non-alcoholic fatty liver disease, inflammatory bowel disease, Crohn’s disease, ulcerative colitis, intestinal permeability, intestinal immune function, gut dysbiosis, gut microbiota-derived metabolites

## Abstract

Despite considerable epidemiological evidence indicating comorbidity between metabolic disorders, such as obesity, type 2 diabetes, and non-alcoholic fatty liver disease, and inflammatory bowel diseases (IBD), such as Crohn’s disease and ulcerative colitis, as well as common pathophysiological features shared by these two categories of diseases, the relationship between their pathogenesis at molecular levels are not well described. Intestinal barrier dysfunction is a characteristic pathological feature of IBD, which also plays causal roles in the pathogenesis of chronic inflammatory metabolic disorders. Increased intestinal permeability is associated with a pro-inflammatory response of the intestinal immune system, possibly leading to the development of both diseases. In addition, dysregulated interactions between the gut microbiota and the host immunity have been found to contribute to immune-mediated disorders including the two diseases. In connection with disrupted gut microbial composition, alterations in gut microbiota-derived metabolites have also been shown to be closely related to the pathogeneses of both diseases. Focusing on these prominent pathophysiological features observed in both metabolic disorders and IBD, this review highlights and summarizes the molecular risk factors that may link between the pathogeneses of the two diseases, which is aimed at providing a comprehensive understanding of molecular mechanisms underlying their comorbidity.

## 1. Introduction

Metabolic disorders are characterized by a chronic inflammatory state that is accompanied by metabolic dysfunctions, such as obesity, insulin resistance, atherogenic dyslipidemia, and non-alcoholic fatty liver diseases (NAFLD). Inflammatory bowel disease (IBD) is a term for conditions that are characterized by chronic inflammation of the gastrointestinal (GI) tract including Crohn’s disease (CD) and ulcerative colitis (UC). Despite a common feature of chronic inflammation shared by these two diseases, the pathogenic association between them is still not clearly understood. Although many epidemiological studies have suggested the possibility of an association between these two types of chronic inflammatory conditions, it is also true that there are still many reports that do not acknowledge the association. One of the reasons for such controversy is that epidemiological studies conducted on different cohorts of subjects may lead to different conclusions regarding the link between metabolic disorder and IBD because the factors affecting the characteristics of a cohort vary widely, causing confusion or hindrance in finding the real link. Therefore, in order to understand more precisely the pathophysiological interaction between the two diseases, an approach that explores the link at the molecular level should be used. In this review, we focus, not only on molecules commonly involved in the pathogenesis of both diseases, but also on molecules specifically related to each disease process to comprehensively understand the molecular networks that connect the risk factors for the two diseases. Given that the risk factors shared by metabolic diseases and IBD include intestinal barrier dysfunction and microbiota dysbiosis, the molecular-level connections related to loss of barrier integrity, intestinal pro-inflammatory state, and alterations in gut microbiota and microbiota-derived metabolites will be discussed in detail. The possible molecular and pathophysiological links between the two diseases described through this review may have potential to be translated into clinical practice where their targeted therapy could improve the efficacy of treatment of IBD patients with comorbid metabolic disorders or vice versa.

## 2. Epidemiologic Interaction between Metabolic Disorders and IBD

Based on the results of many epidemiological studies that have revealed correlations between metabolic disorders and IBD, the causal relationship and shared risk factors between the two diseases have been actively discussed [1,2,3,4,5,6]. Here, we summarize epidemiological and clinical evidence reported until recently in regard to common pathophysiological correlations of the major subtypes of metabolic disorders with IBD.

### 2.1. Metabolic Syndrome-IBD

Recently, evidence has suggested that there is a comorbidity relationship between metabolic syndrome and IBD and these two diseases share common pathogenetic and therapeutic aspects [3]. Two independent studies have demonstrated that the prevalence of metabolic syndrome with increasing age was significantly higher in UC patients compared to CD patients [7,8]. However, the causal relationship between metabolic syndrome and IBD is still unclear because there is also data showing that the frequency of metabolic syndrome in patients with IBD appeared to be comparable to that of healthy controls [7,9]. Rather, some studies have provided evidence that patients with metabolic syndrome had milder form of UC [10]. Considering that the pathogenesis of metabolic syndrome is complicated because it is a collection of multiple metabolic disorders such as obesity, insulin resistance, and atherogenic dyslipidemia, it is understandable that a direct association between metabolic syndrome and IBD has not been clearly observed in cross-sectional studies. As expected, epidemiologic studies on the prevalence of each risk factors constituting the metabolic syndrome in IBD patients have suggested significant associations between them as shown below.

### 2.2. Obesity-IBD

Given that the incidence and prevalence of IBD is rising globally in parallel with the obesity epidemic and there are several risk factors common to both conditions, an association of obesity with IBD has been postulated [1]. Although not all relevant studies showed a complete correlation, it has been shown that body mass index (BMI) and/or increased dietary intake of high fat are clearly associated with increased IBD risk [2]. Etiologic links demonstrating positive associations between obesity and IBD include the dietary composition of Western diet [11,12], gut microbiota alteration [13,14,15], and chronic inflammatory state [16,17,18,19].

### 2.3. Diabetes-IBD

Given the fact that the intestine regulates glucose homeostasis, it has been postulated that chronic intestinal inflammation affects risk for diabetes mellitus, and multiple studies have demonstrated a potential interaction between the two conditions. While some studies have reported that there was no significant association between IBD and type 1 (T1D) or type 2 diabetes (T2D) [20,21], several recent epidemiological studies have shown associations of T1D [22] and/or T2D with IBD [4,23,24,25]. Multiple studies showing the relationship between T1D and IBD (especially UC), in particular, have suggested the genetic association between the two diseases from the findings of sharing of common genetic variants [26,27,28]. Several studies demonstrating the comorbidity of T2D and IBD have suggested that the two conditions could be linked through gut microbiota dysbiosis, epithelial barrier dysfunction, and inflammation [29]. Even though epidemiologic data are still insufficient to confirm the causal relationship between T2D and IBD, many researchers have investigated the nature of the links between the two diseases as they appear to share a common basis influenced by inflammatory processes, which will be discussed later in this review.

### 2.4. NAFLD-IBD

The concomitance of NAFLD among patients with IBD have also been frequently reported [5,6,30,31,32]. Cross-sectional studies have shown elevated levels of aminotransferases in IBD patients, which was suggested to be mainly due to NAFLD (especially hepatic steatosis) caused by overweight or steroid treatment [33,34]. However, the coexistence of NAFLD and IBD appears to be complicatedly influenced by disease-specific risk factors such as metabolic syndrome, chronic inflammation, medication-induced hepatotoxicity, and gut microbiota dysbiosis [35,36]. Although multiple factors in the pathogenesis of IBD and NAFLD have been proposed to explain high NAFLD prevalence in IBD patients, the underlying NAFLD pathogenic IBD-related factors have remained to be elucidated [5,6].

## 3. Metabolic-Endocrine Link between Metabolic Disorders and IBD

Recent data have led to an improved understanding of the association between metabolic disorders and IBD, showing that the pathology of these diseases share common features, including adipose tissue dysregulation, inadequate immune response, and inflammation [37]. Particularly, numerous studies have focused on the role of adipose tissue in the development of metabolic disorders and IBD. As a central metabolic organ for integration and control of whole-body energy homeostasis, the adipose tissue has emerged as an important endocrine regulator that secretes cytokines and hormones, referred to as adipokines, which have pro- or anti-inflammatory activities [38]. While the balance between pro- and anti-inflammatory adipokines maintains the homeostasis in normal metabolic status, this balance is shifted toward a pro-inflammatory state under pathological conditions of dysregulated energy homeostasis, leading to systemic low-grade chronic inflammation, which is associated with an increased risk of obesity, T2D, and cardiovascular diseases [39]. The prevalence of obesity and its metabolic comorbidities is positively correlated with augmented production of pro-inflammatory adipokines such as resistin, visfatin, and chemerin, which is resulted from the expansion of dysfunctional adipose tissue [37,40]. In contrast, the levels of anti-inflammatory adipokines such as adiponectin and omentin-1 are inversely related to obesity and its related metabolic disorders [40,41,42]. In IBD, there is substantial evidence for the involvement of adipose tissue in the intestinal inflammation, in which some adipokines are responsible for the immune modulatory function [43]. It has been reported that serum levels of pro-inflammatory adipokines, resistin and visfatin, were significantly higher in patients with CD or UC than in healthy controls [44,45], whereas, conversely, the levels of adiponectin, leptin, and omentin-1 were negatively correlated with IBD [45,46,47]. The result on the relationship between serum leptin level and disease activity in patients with IBD varies depending on studies, though the reason for the discrepancy remains unclear [45]. Given that adipose tissue is an endocrine metabolic organ and closely interacts with immune system, its crosstalk with intestine mediated by adipokines is of particular interest for exploring the common links between metabolic disorders and IBD.

Changes in enteroendocrine functions also have been implicated in the pathogenesis of both metabolic disorders and IBD. Glucagon-like peptide-1 (GLP-1), an incretin hormone mainly secreted from intestinal L cells, has gained attention as a key player in the pathogenesis of metabolic and inflammatory diseases due to its function in modulating ER stress and promoting anti-inflammatory signaling [48]. The insulinotropic and glucose-lowering effects of GLP-1 has long been shown to be impaired in obesity and T2D [49], and GLP-2, another co-secreted L-cell peptide hormone that improves gut epithelial proliferation and mucosal integrity, has also been found to be reduced in obese mice and humans [50,51]. Secretion of GLP-1 and GLP-2 also has been found to be impaired by chronic exposure to TNFα [52,53] and, inversely, it was reported that GLP-1 or GLP-1 receptor (GLP-1R) agonists reduce the levels of pro-inflammatory cytokines [54] and the activation of GLP-1R signaling modulates intestinal immune responses and mucosal inflammation [55]. Increased levels of circulating dipeptidyl peptidase-4 (DPP4), the enzyme that cleaves GLP-1 and GLP-2, has been demonstrated to be associated with both T2D and obesity [56]. Thus, together with therapeutic applications of GLP-1 and related analogs, inhibitors for the dipeptidyl peptidase-4 (DPP-4), the enzyme that cleaves GLP-1 and GLP-2, and GLP-1R agonists that have a prolonged half-life due to reduced degradation by DPP-4 are currently being used for the treatment of T2D and obesity [57]. On the other hand, studies have shown that serum levels of GLP-1 and GLP-2 were higher in IBD patients than in healthy controls [58], which might be due to an adoptive response to intestinal injury. A study demonstrated that IBD patients had deregulated expression of GLP-1R, chemokine ligand 20 (CCL20), and IL-33 in colon, and this was reversed by the treatment of GLP-1R agonists that led to reduced colonic inflammation [59]. Circulating DPP-4 levels in IBD patients was shown to be lower in comparison with healthy subjects [60], but the link between DPP-4 inhibitors and IBD is not clear since several studies have shown contrary results that DPP-4 inhibitors reduce the risk of IBD among patients with T2D, which reflects their activity of inhibition of T-cell proliferation and cytokine production leading to a GLP-1-mediated restoration of gut mucosal damage [61]. Based on the fact that GLP-1 and GLP-2 improve the intestinal epithelial barrier integrity and regulate mucosal innate immunity, GLP-1, GLP-2, and related analogs have been suggested potential therapeutic options for IBD [58]. A very recent study reported a lower risk of adverse clinical events in patients with IBD and T2D treated with GLP-1R agonists/DPP-4 inhibitors, suggesting the effectiveness of GLP-1 based therapies as treatment for comorbidity of the two diseases [62].

Chronic disorders including obesity, T2D, cardiovascular diseases, and IBD share common features in their pathology, and most importantly, all of them are characterized by chronic inflammation. As summarized above, metabolic-endocrine networks, in which adipose tissue and enteroendocrine cells involved, are highly integrated with the pathogenesis of both metabolic disorders and IBD, and thus have remained at the center of the study of how these diseases begin and progress (a summary of selected studies is shown in Table 1). Dysregulation of the signaling pathways of adipokines and gut hormones induced by impaired functions of adipose tissue and the intestine coexists typically with chronic inflammatory signaling. In particular, chronic inflammation in the intestine is, not only the central pathophysiologic mechanism of IBD, but also related to the pathogenesis of obesity and its associated metabolic complications [63]. The development of obesity-related disorders is strongly related to increased levels in pro-inflammatory cytokines such as TNFα and IL-6, the production of which is also elevated in the intestinal mucosa of patients with IBD [64]. The aberrant shift in intestinal immunity to a pro-inflammatory state induced by metabolic disorders or IBD is closely associated with intestinal barrier dysfunction and/or gut microbiota dysbiosis, which is the main focus of this review and will be discussed in detail in the following sections.

## 4. Intestinal Permeability and Inflammation

### 4.1. Homeostasis of the Intestinal Barrier Function

The concomitance of NAFLD among patients with IBD have also been frequently reported [5,6,30,31,32]. A functional intestinal barrier consists of the mucus layer covering the intestinal epithelial cells (IECs), protecting against both pathogenic and commensal bacteria in the lumen. Underneath the epithelium is the lamina propria, a loose connective tissue containing Peyer’s patches and the cells of immune system including T cells (CD4^+^ and CD8^+^), B cells, and a variety of innate immune cells such as macrophages, dendritic cells (DCs), and eosinophils, as well as innate lymphoid cells (ILCs). Factors in mucosal secretions such as antimicrobial peptides (AMPs) secreted by Paneth cells and secretory immunoglobulin A (sIgA) synthesized in Peyer’s patches also provide another form of protection [107]. Peyer’s patches, which are essential lymphoid organs for the generation of T cell-dependent IgA, also play a critical role in gut homeostasis through regulation of RAR-related orphan receptor γt^+^ (RORγt^+^) cells including group 3 innate lymphoid cells (ILC3s) and T helper 17 (Th17) cells, which produce lymphotoxins, IL-17 and IL-22 and promote intestinal barrier integrity [108]. The intestinal ILC3s and Th17 cells respond mainly to IL-6 and IL-23p19 produced by CD11c^+^ DCs to produce IL-17 and IL-22 by sensing of commensal bacteria through Mincle and Syk signaling [109] and regulate the production of antimicrobial peptide and IgA to prevent the translocation of commensal bacteria across the epithelial barrier and systemic inflammation [110,111]. In addition to these findings, mechanisms of the regulation of intestinal homeostasis and inflammation have been intensively studied focusing on how they are involved in the development of chronic inflammatory diseases. The evidence from those studies has shown that dysfunctions in intestinal barrier integrity and intestinal immunity are very closely related to the pathogenesis of both metabolic disorders and IBD. The following sections (and Table 1) summarize the results of selected recent studies on this topic.

### 4.2. Intestinal Barrier Dysfunctions Induced by Metabolic Disorders

It has long been believed that intestinal barrier dysfunction disturbs intestinal homeostasis allowing the entry of microbial products into the systemic circulation and thereby leads to the development of chronic inflammatory metabolic diseases [66]. Disruption of the intestinal epithelial barrier increases the influx of bacteria and their products into the bloodstream leading to chronic inflammation. Increased translocation of microbiota-derived lipopolysaccharide (LPS) results in a condition of metabolic endotoxemia that is characterized by low-grade inflammation and insulin resistance, and consequently promotes metabolic abnormalities [65,112]. A number of studies have considered altered intestinal permeability as a causative mechanism for metabolic diseases such as T2D, obesity, NAFLD, and non-alcoholic steatohepatitis (NASH) [66,68,69,70,113]. In particular, excess intake of high-fat Western diet has been found to be associated with changes in gut barrier function and microbiota composition, leading to metabolic endotoxemia-triggered obesity and insulin resistance [68,114,115]. However, these associations are not so clear in some studies, demonstrating that asymptomatic obese individuals with low-grade inflammation do not have evidence of gut barrier alteration [116] and gut dysbiosis-induced endotoxemia is not required in the pathogenesis of NASH [117]. Recently, new findings have demonstrated that hyperglycemia drives intestinal barrier permeability and alters tight and adherence junction integrity [118]. In short, although it is clear that an increase in intestinal permeability is commonly associated with metabolic disorders, the question still remains open whether it is a cause or a downstream result.

As for the mechanisms that trigger intestinal barrier dysfunction caused by metabolic disorders, the followings have been intensively studied: reduction in tight junction proteins, dysbiosis of the gut microbiota, and dysregulation of intestinal immune system. As mentioned above, alterations of tight junction proteins, including transmembrane proteins such as claudins and occludin, and peripheral membrane proteins such as zonula occludens (ZOs), can lead to barrier dysfunctions. Several studies have shown the association of a dysfunction of tight junction proteins with metabolic disorders, which was evidenced by decreased expression of ZO-1 and/or occludin or their abnormal distribution leading to increased intestinal permeability in rodent models of high-fat (HF) diet-induced obesity (DIO) and obese human subjects [50,65,66,67,68,119]. The loss of barrier integrity also has been found to be linked to metabolic disorder-associated alterations of the gut microbiota. Both dietary obese and *ob/ob* mice showed that obesity-induced dysbiosis and intestinal permeability could be ameliorated by antibiotic treatment [65], indicating that gut microbiota and their metabolic products have a considerable effect on the function of epithelial barrier. Especially, gut microbiota-derived short-chain fatty acids (SCFAs) have been shown to protect intestinal barrier function through up-regulating tight junction protein and/or inhibiting inflammasome [120,121]. In addition, much of the research focus also has been on molecular immunological mechanisms related to an increased permeability of the intestinal barrier and disturbed immune homeostasis, which are summarized in next section.

### 4.3. Intestinal Pro-Inflammatory State Induced by Metabolic Disorders

The intestinal barrier dysfunction induced by metabolic disorders is directly related to the shift of the intestinal immune system toward a pro-inflammatory state. Many studies have shown that HF diet (HFD)-induced metabolic disturbance is associated with intestinal chronic inflammation characterized by the increased levels of pro-inflammatory cytokines [63]. That is, obesity and insulin resistance induced by HFD are associated with low-grade inflammation in the intestine accompanied by increased levels of pro-inflammatory cytokines such as TNFα, IL-1β, IL-6, and IL-12. As a causative mechanism for this upregulation of pro-inflammatory cytokines, studies have focused on the interaction between the intestinal microbiota and immune system. Increased intestinal permeability due to abnormal barrier function associated with metabolic disorders results in increases in the binding of microbial-associated molecular patterns (MAMPs) to pattern recognition receptors (PRRs), such as toll-like receptors (TLRs), and nucleotide-binding oligomerization domain (NOD)-like receptors (NLRs), which promotes the release of pro-inflammatory cytokines from intestinal epithelial cells [86]. Secreted cytokines, in turn, stimulate the secretion of other pro-inflammatory cytokines including IL-6, IL-12, and IL-23 by innate immune cells such as macrophages and DCs that induce development of the effector CD4^+^ Th1 and Th17 cells. Beyond these findings, it also has been shown that the association of HFD-induced obesity with increased intestinal permeability is related to pro-inflammatory shift in intestinal immune cells, including a decrease in regulatory T cells (Tregs), increases in IL-17-producing γδ T cells, IFN-γ-producing Th1 and CD8^+^ T cells [87,88]. This is further supported by the previous findings that IFN-γ and other pro-inflammatory cytokines increase the intestinal permeability through direct perturbation of the tight junction proteins [71,87]. In contrast, IL-22 was found to have a protective effect against intestinal barrier dysfunction. Various types of obese (*db/db*, *ob/ob*, and DIO) mice showed a dramatic reduction in IL-22 production from colonic innate lymphoid cells (ILCs) and Tregs upon infection with enteric pathogen *Citrobacter rodentium*, and IL-22 treatment reversed the endotoxemia-induced chronic inflammation and insulin resistance [89]. In addition, the balance between Th17 and Treg cells in the intestine is also closely connected with the pathogenesis of obesity and related metabolic disorders. Many studies have shown that the Th17/Treg balance is regulated by inflammatory cytokines and various metabolic factors, as well as interaction with gut microbiota, and its alteration has important roles in the development of chronic inflammatory metabolic disorders such as obesity, T2D, and NAFLD [85]. Besides, HF-induced depletion of intestinal eosinophils was also demonstrated to lead to a loss of barrier integrity in DIO mice, but the underlying mechanism needs to be clarified [91].

### 4.4. Intestinal Barrier Dysfunctions Induced by IBD

IBDs are chronic inflammatory conditions of the gastrointestinal tract that are characterized by disrupted intestinal homeostasis and caused by a combination of genetic susceptibility, inappropriate immune responses to the luminal microbiota, and exposure to environmental factors. Although the mechanisms underlying the pathogenesis of IBD are not yet fully understood and UC and CD, the two major types of IBD, have different mechanisms of tissue damage, enough evidence has been accumulated for the role of the intestinal barrier dysfunctions in both the subtypes of IBD. That is, the disruption of intestinal epithelium integrity is thought to be an early event in IBD pathogenesis, allowing bacteria and their products to penetrate through a barrier leak leading to aberrant immune response and inflammation. Altered expression of proteins regulating tight junctions might be the one of the molecular mechanisms underlying the loss of barrier integrity and increased intestinal permeability observed in IBD patients [122]. Among tight junction proteins, claudins have been focused and studied most intensively. It has been known that increased expression of pore-sealing claudins (e.g., claudin-3, -4, -5, -7 and -8) leads to increased number of tight junction strands, consequently decreasing barrier permeability, and in contrast, claudin-2 and -13 decrease epithelial tightness and increase solute permeability by forming cation-selective and water channels [73]. Multiple studies have confirmed the pathological modification of claudin expression in IBD patients, i.e., the downregulation of pore-sealing claudin-4, -5, and -8 and upregulation of pore-forming claudin-2 [71,72,74,122]. In the case of occludin, several studies demonstrated its decreased expression in both UC and CD compared to healthy controls [72,75], but in another study there was no difference [74]. Reduced expression of ZO-1 has been displayed in cultured human colonic epithelial cells [72] and DSS-induced colitis mice [75,76]. In addition to the altered expression of tight junction proteins, genetic susceptibility may also be related to increased risk of IBD. It has been revealed that polymorphisms of genes encoding tight junction-associated proteins such as myosin IXB (*MYO9B*), partition-defective 3 (*PARD3*), membrane-associated guanylate kinase inverted 2 (*MAGI2*), guanine nucleotide-binding protein α12 (*GNA12*) and protein tyrosine phosphatase TCPTP (*PTPN2*), cell adhesion-related proteins such as E-cadherin (*CDH1*) and laminin β1 (*LAMB1*), and mucus layer proteins such as mucin 3A and 19, are associated with the risk of IBD [77,78].

Intestinal barrier dysfunction may also be caused by epithelial damage including pathological intestinal cell shedding and/or cell death, which interplays with innate immunity in intestinal inflammation [79]. At the end of the life cycle (3–5 days), IECs shed from epithelium. Since cell death in the intestinal barrier is finely controlled and epithelial cell shedding does not require cell death, the shedding-associated apoptosis might be a consequence rather than a cause of the shedding process. Recent investigations have shown that the activation of caspases can be seen mainly in cells that are already in shedding, indicating that caspase-mediated apoptotic cell death is not a prerequisite for epithelial cell shedding [123]. However, these observations do not mean that apoptosis does not cause IBD. Several studies have reported that dysregulated or excessive apoptosis in the intestinal epithelium indicate that gut inflammation is more likely to develop [80,81]. Molecular factors connecting epithelial apoptosis and intestinal inflammation include NF-κB, REL-A, transforming growth factor-activated kinase (TAK)1, and IκB kinases in IECs, which could increase the susceptibility to IBD [82]. Necrosis, an accidental and uncontrolled cell death, and necroptosis, a new caspase-independent programmed cell death, have also been suggested to play a role in the pathogenesis of IBD [79]. Both necrosis and necroptosis show similar morphological features and are characterized by the release of cytoplasmic content into extracellular space leading to the activation of PRRs such as TLRs, which triggers inflammatory responses [83,84].

### 4.5. Intestinal Pro-Inflammatory State Induced by IBD

An abnormal immune response to microbiota in intestinal mucosa has been well-known to be responsible for IBD pathogenesis. Recent advances in IBD research have provided evidence that the pathogenesis of IBD is associated with a dysregulated immune response of CD4^+^ T cells and ILCs. Multiple subsets of CD4^+^ T cells have been found to mediate diverse protective and homeostatic responses, and perturbations of their functions may lead to chronic intestinal inflammation. CD4^+^ T cells are present at increased frequencies in inflamed tissue of patients with IBD, and a number of drugs targeting cytokines involved in CD4^+^ T cell differentiation into inflammatory subsets have shown clinical efficacy in treating IBD patients [124]. After being stimulated by antigens, naïve CD4^+^ T cells are activated and differentiate into distinct subsets of effector cells including T helper type 1 (Th1), Th2, Th17, Th22, T follicular helper (Tfh), and regulatory T (Treg) cells, which play different roles in mediating immune response via the secretion of characteristic inflammatory cytokines such as IFN-γ, IL-4, IL-9, IL-17, IL-22, IL-21, and IL-10/TGF-β, respectively [95]. A hallmark of the immunopathology of IBD is the dysregulation of Th1, Th2, Th17, and Treg cell responses that leads to the imbalance between Th1/Th2 and Th17/Treg cells and alters intestinal homeostasis [92,125]. In early studies on how each effector T cell subset functions in the development of IBD, Th1 or Th2 responses were focused and described, with the former being dominated by the production of IFN-γ and the latter by the secretion of IL-4 and IL-5. Several studies have clearly shown that CD patients have a higher frequency of Th1 cells secreting IFN-γ in the inflamed mucosa, and that elevated Th2 or Th2-like responses are more associated with the pathology of UC [96,97]. In addition to Th1 and Th2 cells, another T helper subset, termed Th17 cells, have recently been discovered to be the most notable subtype of CD4^+^ T cells playing a crucial role in IBD pathogenesis [126]. Th17 cells are differentiated from naïve T cells in the presence of IL-6 and TGFβ and maintained by accessory cytokines IL-1β and IL-23 [127]. Th17 cells and their producing cytokines, IL-17, IL-21, and IL-22 have been observed to be present at increased levels in the inflamed intestine of active IBD patients [99]. Moreover, it has been demonstrated that CD is associated with either Th1 or Th17 cell-mediated response induced by IL-12 and IL-23, producing IFN-γ and IL-17 as pro-inflammatory signals, whereas UC is associated with, not only the abundance of Th17 cells, but also an atypical Th2-mediated response resulting in the expansion of NKT cells producing IL-13 and increased production of IL-4 and IL-5 [98,100]. As such, it is clear that massively infiltrated Th17 cells producing Th17-related cytokines in excess play a key role in IBD pathogenesis, but the roles of T cell plasticity, particularly Th1/Th17 or Treg/Th17 balances also could not be ruled out [93,94]. Naïve CD4^+^ T cells can also differentiate into Treg cells, which suppress excessive inflammation to maintain intestinal immune homeostasis [95]. In IBD, CD4^+^ Treg cells can convert into Th17- or Th1-like cells that then contribute to inflammation, which means that the balances between Treg and T17 or Th1 cells are critical [128]. In the inflamed intestinal mucosa of CD patients, Th17 cells differentiate into Th1-like Th17 (Th17/Th1) cells that produce both IFN-γ and IL-17 (IFN-γ^+^ IL-17^+^) and play a strong pathogenic role, and also Treg cells convert to FoxP3^+^ IL-17^+^ (Treg/T17) cells with reduced suppressive function [101]. More recently, with evidence of accumulation of IFN-γ-producing cells in the inflamed tissue of both CD and UC patients, it was demonstrated that Th1-like Treg (Th1/Treg) cells are also required for the development of intestinal inflammation, especially Th1-mediated colitis [102].

Innate lymphoid cells (ILCs), which are recently identified group of innate immune cells that share similarities with T lymphocytes but lack antigen-specific receptors, are also implicated as regulators of chronic intestinal inflammation, playing crucial roles in the pathogenesis of IBD. Based on their transcription factor requirement and cytokine production profiles, they have been classified into three main groups: group 1 ILCs (ILC1) that produce the Th1-like cytokine IFN-γ under the control of transcriptional factor T-bet; group 2 ILCs (ILC2) that secrete Th2 cytokines such as IL-5, IL-9, and IL-13 in the presence of GATA3; and group 3 ILCs (ILC3) produce Th17/Th22-like cytokines IL-17 and IL-22 in the presence of the RORγt, and additionally on the basis of the expression of natural cytotoxicity receptors (NCRs) including NKp46 and NKp44, ILC1s and ILC3s can be subdivided into NCR^+^ and NCR^−^ subsets [103]. An increasing body of evidence indicates that ILC3s, in particular, play a critical role in maintaining intestinal mucosal homeostasis [105]. In inflamed epithelium, upon exposure to IL-12 produced by DCs, NKp44^+^NKp46^+^ ILC3s can be transdifferentiated into NKp44^−^NKp46^−^ IFN-γ-producing ILC1s through up-regulation of T-bet with a concomitant downregulation of RORγt. This plasticity of ILC3s toward ILC1s underlies the observation that, in the inflamed intestine of CD patients, ILC1 markedly accumulate enough to be the predominant subset accompanied by a decreased number of IL-22-producing ILC3s [103,104]. In addition, the plasticity of ILC2s to ILC1s driven by IL-12 has also been found to be associated with the pathology of CD [129]. Regulatory ILCs (ILCreg), a recently identified subset of ILCs, which appear to be distinct from Treg cells due to the lack of expression of CD4 and FoxP3 [130], have also been found to contribute to maintaining intestinal homeostasis and protecting against inflammation through generating IL-10 and TGF-β, thereby suppressing the production of IFN-γ and IL-17A by ILC1 and NCR^−^ ILC3, respectively [105]. Dysregulated activation of ILCregs could be linked to an impairment of regulatory anti-inflammatory function in the innate immunity, leading to the development of IBD [106].

## 5. Gut Microbiota Dysbiosis

In recent years, the gut microbiota has emerged as a critical contributor to the control of host metabolism and immune functions. Over the past two decades, numerous studies have shown that the interactions of the intestinal microbiota and immune system regulate the function of the innate and adaptive immunity and the dysregulation of microbiota-immunity interaction may contribute to the development of immune-mediated diseases including IBD, metabolic disorders, cancer, and rheumatoid arthritis [131]. Now, it is a well-established fact that alterations in the gut microbiota and their metabolites are associated with the development and progression of major chronic inflammatory diseases including metabolic disorders and IBD [132,133,134].

### 5.1. Interaction between Intestinal Microbiota and Immunity

The intestinal microbiota interacts with the innate and adaptive immune systems in a variety of ways. In relation to the intestinal innate immunity, endogenous antimicrobial peptides (AMPs) released by Paneth cells at mucosal surfaces are essential components in mediating innate immune responses [135]. They, including defensins and cathelicidins, control the composition of the commensal microbiota and play a key role in intestinal barrier function and maintaining epithelial homeostasis. Recognition of pathogen-associated molecular patterns (PAMPs) by epithelial cells through TLRs is also an integral part of innate immunity. TLRs mediate host defense against microbial pathogens and maintain intestinal homeostasis by balancing the pro- and anti-inflammatory immune responses to both commensal and pathogenic microbiota [136]. In particular, TLR5, the receptor for bacterial flagellin, has been shown to play an essential role in the maintenance of intestinal barrier function through elimination of microbial pathogens. As with TLRs, activation of NLRs also has been shown to contribute to shaping the composition of the microbiota. Among NLRs, NOD1 and NOD2 are critical for innate defense serving as intracellular receptors for bacterial peptidoglycan fragments [137,138]. Several inflammasomes, such as NLRP3, NLRP6, and the absent in melanoma 2 (AIM2), also have roles in defense against infections with traditional pathogens and control of the intestinal microbiota [139]. As mentioned in the previous section, innate immune effector cells such as macrophages, DCs, monocytes, and ILCs are certainly involved in maintaining homeostasis between the intestinal microbiota and the mucosal immune system.

Gut microbiota mutually communicates, not only with innate immune function, but also with adaptive immunity. Many studies have found that Foxp3^+^ RORγt^+^ Treg cells mediate immune tolerance to the microbiota [140], and their induction and maintenance critically depend on the microbiota [141,142,143]. Specialized Foxp3^+^ T follicular regulatory (Tfr) cells were also suggested to contribute to maintenance of diversified and balanced microbiota through regulation of IgA selection in Peyer’s patches [144]. The colonization of commensal segmented filamentous bacteria (SFB) was found to induce RORγt^+^ Th17 cells to produce IL-17A, which is mediated by ILC3-derived IL-22 [145], as well as promote the differentiation of Peyer’s patch Tfh cells [146]. The differentiation of CD4^+^ Th17 cells, which have been known to have defensive functions against bacterial pathogens, were also demonstrated to be induced in response to colonization with SFB and certain extracellular pathogens [147]. Intestinal DCs also respond to commensal bacteria, producing IL-6 and Il-23p19, thereby induces the production of IL-17 and IL-22 by Th17 and ILCs, which enhances intestinal barrier integrity [109]. Furthermore, it has been found that microbiota-derived SCFAs can regulate adaptive immune responses through their effects on a number of cell types, including CD4^+^ T cells [148,149]. Particularly, butyrate has been reported to induce IL-10^+^ T cells and FoxP3^+^ T cells by activating its receptor GPR109A on macrophages and dendritic cells [150] or suppressing histone deacetylases in CD4^+^ T cells [151]. Gut microbiota-derived metabolites other than SCFAs, including bile acids, trimethylamine N-oxide (TMAO), branched-chain amino acids (BCAAs), tryptophan and indole derivatives, are also implicated in intestinal and systemic metabolic homeostasis (see below).

### 5.2. Alterations in Gut Microbiota and Microbiota-Derived Metabolites in Metabolic Disorders

Numerous studies have confirmed that intestinal microbiota dysbiosis causes abnormal inflammatory responses and alters the functions of adaptive immunity leading to metabolic disorders. The interplay between the intestinal microbiome and immune system has been demonstrated to play an important role in the pathogenesis of obesity and insulin resistance, which are associated with chronic inflammation in metabolic tissues such as adipose tissue and the liver [63]. Accumulating evidence reveals that metabolic disorders are characterized by altered microbiota composition and microbiota-derived metabolites, being involved in the activation of metabolic inflammation [152]. Notably, intestinal microbiome richness correlates with obesity-related complications such as insulin resistance and dyslipidemia, and lower microbial gene richness present more severe metabolic dysfunctions and chronic inflammation [14,153]. In a recently reported study, an altered microbiome composition was shown to promote the post-dieting weight gain and metabolic aberrations in obesity [154]. Over the past 15 years, a number of studies have identified distinct signatures of gut microbiome associated with obesity, which is summarized in Table 2 [155,156,157,158,159,160,161,162].

Development of T2D also has been linked to altered microbiome composition,; for example, a metagenome-wide association study analysis suggested an altered composition of the gut microbiota that was characterized by a decreased abundance of certain butyrate-producing bacteria including *Faecalibacterium prausnitzii*, *Roseburia intestinalis,* and *Roseburia inulinivorans*, and an increased various opportunistic pathogens including *Clostridium* species, in patients with T2D [163], which was similar to another reported gut microbiome signature observed in the fecal metagenome of European T2D women [164]. The association of gut dysbiosis with insulin resistance is further verified by the observation that when the intestinal microbiota from lean donors was transferred to individuals with metabolic syndrome, insulin sensitivity was recovered [201]. The gut microbiome signature for T2D may also include a decreased abundance of a mucin-degrading bacterium *Akkermansia muciniphila*, which can be considered together with the data showing an improved glucose tolerance when it was treated to in diabetic rodents and human subjects [90,173], and an increased abundance of the main species driving the association between biosynthesis of BCAAs and insulin resistance, *Prevotella copri* and *Bacteroides vulgatus* [202]. In addition to these, there have been many reports on distinct gut microbiome dysbiosis in T2D (a summary of selected studies is shown in Table 2), some of which are commonly associated with obesity [165,166,167,168,169,170,171,172,174,203]. Besides, apart from dysbiosis signatures of gut microbiota, microbial signatures in metabolic tissues such as adipose tissue and the liver have recently emerged as a new research focus. Increased intestinal permeability allows the translocation of gut bacteria into the blood and then to metabolic organs, contributing to chronic inflammation and development of T2D. Several studies have provided evidence for the presence of tissue-specific microbial signature in obesity or T2D [204,205], for example, a reduced bacterial diversity concomitant with decreased abundance of *Faecalibacterium* and increased abundance of Enterobacteriaceae especially in mesenteric adipose tissue of patients with T2D [174].

In NAFLD, the gut microbiota dysbiosis has been known to be implicated as a factor in pathogenesis. Like obesity and T2D, a number of studies have reported the distinct compositions of the gut microbiota in animal models and human patients of NAFLD, the summary of which is included in Table 2 [175,176,177,178,179,180,181,206,207,208,209]. Further, many metagenomics studies in NAFLD have provided evidence for the existence of fecal-microbiome-derived metagenomic signatures that show the degree of NAFLD progression [180,210,211]. Regarding the molecular mechanisms underlying gut dysbiosis-induced pathogenesis, an earlier study on inflammasome-mediated inflammation associated with alterations in the gut microbiota composition has shown that the NLRP6 and NLRP3 inflammasomes and the effector cytokine IL-18 negatively regulate NAFLD progression [212]. In addition to inflammasomes, TLRs are also capable of mediating the inflammatory responses in metabolic disorders. TLR4, the most well-studied TLR, has been known to induce innate immune responses to LPS, which is a PAMP, triggering pro-inflammatory cascades, which plays an important role in the pathogenesis of NAFLD and NASH [213]. As summarized above, disease-specific gut microbiota signatures in obesity, T2D, and NAFLD have been and being identified, some of which are closely related to each other.

In connection with altered intestinal microbial composition, dietary factors and microbiota-derived metabolites have also been shown to be closely related to the pathogenesis of metabolic disorders. Altered signals derived from the diet-microbiome interactions, which are accompanied by the alteration of gut microbiota composition and its function, might contribute to metabolic dysregulation and inflammation [214]. A recently published review on dietary factors that are essential for the relationship between human health and the gut microbiota summarizes the current state of studies on the relationship underlying the effects of diet on metabolic disorders [215]. In addition, there is a wealth of evidence, both in mice and humans, demonstrating that metabolites produced by gut microbiota, such as SCFAs, BAs, TMAO, BCAAs, tryptophan, and indole derivatives, can exert effects on the development of metabolic disorders.

Numerous studies have demonstrated that the most abundant SCFAs, such as acetate, propionate, and butyrate, have beneficial roles in maintaining intestinal barrier integrity and energy metabolism [216,217], and their production is reduced in rodents and humans with metabolic disorders [218,219,220,221,222,223,224] (Table 3). A high-fiber diet improves glycemic control in patients with obesity and T2D via modulation of gut microbiota, which was correlated with altered carbohydrate fermentation and promoted growth of SCFA-producing gut bacteria [222,225]. This beneficial effect has been evidenced by the observations that obese rodents or human subjects supplemented with SCFAs or prebiotic inulin had reduced adiposity, improved glucose control, and/or restored intestinal barrier dysfunctions [219,221,223,224]. Several studies have suggested potentially underlying mechanisms related to the association between changes in SCFA-producing bacteria and the pathogeneses of metabolic disorders. For example, the beneficial role of NLRP12, an inhibitory innate immune sensor, in regulating inflammation was shown through the observation that its reduced expression correlates with decreased Lachnospiraceae family, whose members are well-known SCFA producers, and the associated SCFA synthetic enzymes in HFD-induced obese mice [226]. Another study reported that gut dysbiosis including an aging-associated decrease in beneficial commensal bacteria such as *Akkermansia muciniphila* can lead to insulin resistance, which was evidenced by the findings that the loss of *A. muciniphila* and its product butyrate caused intestinal integrity disruption and endotoxin leakage, thereby activating CCR2^+^ monocytes and in turn triggering 4-1BB receptor signaling of innate B1a cells, resulting in impaired insulin signaling [227]. Despite all this evidence, however, it should be also noted that some studies have reported contradictory results showing that obese mice and human subjects have higher levels of cecal and fecal SCFAs than their lean controls [228].

Gut dysbiosis-induced dysregulation of bile acid metabolism has also been identified to contribute to the pathogenesis of metabolic disorders. Bile acids control bacterial overgrowth in the small intestine, regulate the composition of microbiota, and protect against inflammation by activation of host innate immune responses [262]. Vice versa, gut microbiota can modulate bile acid composition via deconjugation or 7-dehydroxylation of primary bile acids such as cholic and chenodeoxycholic acid, producing secondary bile acids including deoxycholic and lithocholic acid [263,264]. Bile acid deconjugation is catalyzed by bile salt hydrolase (BSH) expressed by a broad spectrum of gut bacteria, whereas 7-dehydroxylation catalyzed by 7α-dehydroxylases is carried out by a limited number of species of *Clostridium* cluster XVIa and XI, the best-characterized of which is *Clostridium scindens* [265,266,267]. Although the levels of circulating bile acids have been demonstrated to be positively correlated with metabolic disorders [229,231,268], it is not clear whether altered bile acid metabolism is the result or cause of disease progression [269]. Several recent studies have shown how disease-associated alterations of gut microbiota and bile acid composition were recovered when obesity-related metabolic disorders were treated [232,233,234,270], a summary of which is included in Table 3. Given that farnesoid X receptor (FXR), the receptor for bile acids, plays a pivotal role in maintaining bile acid, lipid, and glucose homeostasis, it is not surprising that FXR-deficient mice developed NAFLD phenotypes, and patients with metabolic disorders had decreased level of FXR expression in their liver and intestine [271,272]. Furthermore, FXR knockout mice on a diet with excessive fat and sucrose had steatohepatitis and increased levels of free and conjugated secondary bile acids, accompanied by reduced Firmicutes and increased Proteobacteria, which could be reversed by antibiotics treatment [273]. Particularly, it has been demonstrated that the BSH activity of the gut microbiota influences host weight gain, glucose and lipid metabolism, which could be used as a target for the treatment of obesity, T2D, and hypercholesterolemia [230,274].

Trimethylamine-N-oxide (TMAO), another gut microbiota-derived metabolite, is also linked with gut microbiota alteration in metabolic disorders. TMAO is produced by gut microbial metabolism of dietary carnitine and choline and has been found to be involved in the development of cardiometabolic complications [235,275]. Many studies have shown that the level of TMAO positively correlates to the risk and development of T2D and obesity [236,237,239] (Table 3). A recent study suggested a gut microbiota-initiated TMAO-producing pathway that was evidenced by protection of mice against HF diet-induced obesity through pharmacologic and genetic inhibition of flavin-containing monooxygenase 3, the hepatic TMAO-producing enzyme [238].

Tryptophan and indole-derivative metabolites have also been frequently recognized to be related to the metabolic disease-associated gut dysbiosis. Dietary tryptophan can be metabolized via the indole pathway in bacteria to produce indole and its derivatives, some of which serve as ligands for aryl hydrocarbon receptor (AHR), a xenobiotic receptor known to have a vital role in maintaining intestinal barrier integrity [276]. Emerging evidence suggests that the reduced capacity of the microbiota to produce the AHR activating metabolites could be a key factor in metabolic syndrome [242], the mechanism of which might be associated with intestinal barrier dysfunctions caused by decreased production of GLP-1 and IL-22, thereby leading to chronic inflammation, liver steatosis, and insulin resistance [277,278]. A few studies have demonstrated that the activity of indoleamine 2,3-dioxygenase 1 (IDO1), the key enzyme responsible for tryptophan degradation to kynurenine in the extrahepatic organs including the intestine, is increased in adipose tissue of obese patients [240,241] (Table 3). It was also shown that IDO1-deficient mice are protected from obesity, hepatic steatosis, insulin resistance, and chronic inflammation, which is through a shift of tryptophan metabolism from kynurenine pathway to a gut microbiota-mediated production of indole derivative, leading to increased intestinal level of IL-22 and preserved intestinal barrier function [115]. Moreover, tryptophan-derived metabolites produced by the gut microbiota was found to control the expression of the miR-181 family, which promote the development of insulin resistance and WAT inflammation in obese mice and humans, to regulate energy expenditure and insulin sensitivity [243].

In addition, dysregulated metabolism of BCAA such as valine, leucine, and isoleucine are also associated with metabolic disorders. Elevated levels of plasma BCAAs have been demonstrated to have a strong correlation with insulin resistance, and therefore, BCAA was suggested as a biomarker for T2D diagnosis [244,245,246,247] as well as a target for the treatment of obesity-associated insulin resistance [248] and hepatic steatosis [180] (Table 3). It was recently reported that brown fat actively utilizes BCAAs for non-shivering thermogenesis and promotes systemic BCAA clearance, thereby contributing to the improvement of metabolic health [279]. Among the components of human gut microbiota, *Prevotella copri* and *B. vulgatus* were found to be the main species driving the association between biosynthesis of BCAAs and insulin resistance [202].

### 5.3. Alterations in Gut Microbiota and Microbiota-Derived Metabolites in IBD

Many recent studies have identified key roles of the gut microbiota in the pathogenesis of IBD. Data from earlier studies have shown that IBD represents a dysregulated mucosal immune response to the commensal microbiota and a reduced biodiversity of gut bacterial community [182,183,198]. Together with the loss of diversity, gut dysbiosis characterized by decreased abundance of Bacteroidetes [182,184,196,199], Firmicutes [182,184], Verrucomicrobia [199] at phylum level, Clostridia [182] at class level, Erysipelotrichales, Bacteroidales, and Clostridiales [198] at order level, Ruminococcaceae [182,185,186], Akkermansiaceae and Bacteroidaceae [199] at family level, *Bacteroides*, *Lactobacillus*, *Bifidobacterium* [182], *Prevotella, Eubacterium, Odoribacter, Akkermansia, Roseburia, Parabacteroides*, *Alistipes, Coprococcus, Dorea, Ruminococcus* [192], *Akkermansia, Faecalibacterium* and *Bifidobacterium* [199] at genus level, and increased abundance of Proteobacteria [195,199] and Actinobacteria [199] at phylum level, Gammaproteobacteria [182] at class level, Clostridiales [197] at order level, Enterobacteriaceae, Veillonellaceae [198,199], Pasteurellacaea, Fusobacteriaceae [198], Ruminococcaceae [197], Staphylococcaceae, and Streptococcaceae [199] at family level, *Escherichia* [185,192,199], *Klebsiella, Enterococcus*, *Veillonella* [192], *Faecalibacterium, Fusobacterium* [197], *Shigella, Peptostreptococcus, Bacillus,* and *Veillonella* [199] at genus level were also found to be associated with IBD pathogenesis, as shown in a summary of selected studies in Table 2. However, the abundance of certain bacterial taxa does not always have the same meaning as its functional activity. To close the gap between gut microbiota composition and its functional activity, a metagenomic and metatranscriptomic analysis, as part of the integrative Human Microbiome Project (iHMP) [187], was recently performed and the results provided complementary insights linking the metagenomic functional potential to the realized expression of genes in the IBD gut microbial community [188]. Additionally, despite conflicting results for changes in abundance of a few taxa between studies, CD and UC, the two major types of IBD, exhibit similar pattern of gut dysbiosis, but there are also some CD- or UC-specific bacterial taxa. A recent study using high-resolution shotgun metagenomics sequencing, which differs from previous 16S rRNA sequencing studies, showed that 87 of the 102 UC-associated taxa were also found to be associated with CD, and 15 taxa including *Bacteroides uniformis* and *Bifidobacterium bifidum* were associated specifically with UC [185]. Another recent study performed an unbiased meta-analysis of five different IBD datasets available in the public domain and found consistent differences between the gut microbiota of CD and UC patients, suggesting disease specificity in gut microbial community and metabolism for CD versus UC [280].

Owing to extensive studies focusing on the role of microbiota dysbiosis in IBD development, the immunopathogenesis of IBD also has become better understood [281]. Multiple lines of evidence suggest that gut microbiota is an important regulator of epithelial-immune cell communication, especially in shaping the intestinal barrier, and dysregulated immune responses can lead to a disturbance of the microbiota [123]. Very recently, a study has provided evidence for gut dysbiosis attributed to mucus layer defects caused by Muc2 mutation in colitis-prone mice [282]. This pathogenic role of gut dysbiosis in IBD has also been confirmed by the studies showing that various strains of probiotics such as *Lactobacillus rhamnosus*, *Enterococcus faecalis*, *Bifidobaceterium brevis*, *E. coli Nissle* 1917, and VSL#3, a probiotic preparation including *Lactobacillus*, *Bifidobacterium*, and *Streptococcus* strains, display a beneficial effect on the intestinal epithelial barrier function [123,283]. A metagenomic analysis of gut microbiome in patients with IBD revealed a higher abundance of facultative anaerobes capable of tolerating oxidative stress, and of *Ruminococcus gnavus*, a mucolytic bacterium capable of degrading colonic mucin [190], which has been reported to be accompanied by decreased SCFA production and the development of colitis and metabolic syndrome [191]. High abundance of *R. gnavus* was also presented by multi-omics IBD studies that are part of the iHMP [188,189]. The gut dysbiosis of CD or UC is also characterized by lower abundance of *Roseburia hominis* and/or *F. prausnitzii*, both butyrate-producing bacteria belonging to the phylum Firmicutes [182,189,193,194].

A growing number of studies are pointing out the role of, not only disrupted gut microbial composition, but also metabolites derived from altered gut microbiota in the development of IBD. Similar to metabolic disorders, the gut microbiota-derived metabolites implicated in IBD pathogenesis include SCFAs, bile acids, tryptophan metabolites, BCAAs, and TMAO (Table 3). The association of lower levels of SCFAs with increased risk of IBD has been known for over 30 years, and it was recently shown that the reduced butyrate-synthetic capacity of the microbiota was revealed as a hallmark of patients with CD, but less with UC, which might relate to reduced dietary-fiber intake [249]. In the same context, high intake of dietary fiber or diet-derived SCFAs was demonstrated to protect against DSS-induced colitis in mice, the mechanism of which is through SCFAs-mediated activation of G-protein-coupled receptors such as GPR43 and GPR109A, and thereby activating NLRP3 inflammasome [121]. Contrarily, the activation of NLRP1 inflammasome was found to have negative effect on the abundance of beneficial, butyrate-producing Clostridiales through promoting IL-18 and IFN-γ production, which promotes the development of IBD [200]. On the other hand, the butyrate-producing *F. prausnitzii* was reported to prevent colitis in chemically induced IBD model mice by producing a protein with anti-inflammatory properties that inhibits the NF-κB pathway in intestinal epithelial cells [15]. Given that, as described above, gut microbiota-derived SCFAs play an important role in regulating gut homeostasis, thereby preventing the development of metabolic disorders [219], reduced production of SCFAs could be a common risk factor that is associated with both IBD and metabolic disorders. Very recently, my reseach group’s study using DSS-induced colitis model mice has shown that IBD is associated with adipose tissue dysfunction and disrupted hepatic lipid metabolism leading to hepatic steatosis and dyslipidemia [284], which was found to be mediated by a decreased level of SCFAs, being displayed through comparison of SCFA production between germ-free mice transplanted with fecal microbiota of colitis mice and those transplanted with non-colitis mice microbiota [285].

The interaction between bile acids and microbiota also plays a role in IBD pathogenesis. Emerging evidence from metabolomic analysis shows that patients with IBD have depleted levels of secondary bile acids and over-abundance of primary bile acids [250,251]. High-fat diet intake could be associated with the incidence of IBD, which is through a modification of bile acid metabolism that induces pathogenic alterations in gut microbiota composition. For example, it was demonstrated that a high saturated-fat diet led to increased production of taurocholic acid, a taurine-conjugated bile acid, in the liver, which in turn, promoted the growth of a sulphite-reducing pathobiont, *Bilophila wadsworthia*, and triggered colitis in genetically susceptible IL-10-deficient, but not wild-type mice [252]. A metagenomics analysis reported a significantly reduced abundance of Firmicutes phylum-derived BSH gene in fecal bacteria in UC and T2D patients but not in CD patients [230].

In addition, increased tryptophan metabolism has been shown to be negatively correlated with the disease activity of IBD [286]. In an analysis of tryptophan and its metabolites in more than 500 patients with IBD, lower serum level of tryptophan and higher levels of tryptophan metabolites, especially of quinolinic acid, were observed, which indicated a high activity of tryptophan degradation in patients with active IBD [255]. Indole-derivatives produced from tryptophan by microbiota and their receptor AHR have also been found to be implicated in IBD pathogenesis. AHR is known to have a role in modulating intestinal inflammatory responses, thereby exerting protective effects against colitis [287]. AHR activation suppresses inflammation through promoting IL-22 production, and IBD patients have a reduced level of AHR in the intestine [256]. A recent study has found a relationship between gut microbiota and caspase recruitment domain family member 9 (CARD9), a susceptibility gene for IBD involved in recovery from colitis and shown that the microbiota from CARD9-deficient mice failed to convert tryptophan into indole derivatives that activate AHR, the reduced production of which was also observed in the microbiota from human subjects with IBD [257]. Indole-3-propionic acid (IPA), a microbiota-derived indole as an AHR ligand, has been reported that protects from chemically induced colitis in mice, and its level in serum of patients with UC is lower than healthy controls [258]. IPA was suggested to improve colitis through the induction of IL-10R1 on colonic epithelia. Besides, another indole derivative activating AHR, indoleacrylic acid, has been shown to be produced together with IPA by several commensal *Peptostreptococcus* species and improve intestinal barrier function through suppressing pro-inflammatory cytokine production [259].

On the other hand, the association of BCAAs with IBD remains unclear. A recent study to explore the role of disturbed amino acid metabolism in the pathogenesis of CD reported a negative correlation of the plasma levels of leucine and valine, but not other BCAAs, with disease activity in CD patients [260], whereas another study showed no significant correlation of plasma BCAAs with UC [261]. Even though it has been suggested that BCAAs play a positive role in maintaining intestinal barrier function [288], some studies using chemically induced colitis model mice have shown contradictory results, and it is also contrary to the association of higher BCAA levels with T2D [133]. Similar to BCAAs, association between circulating TMAO and IBD has not been well identified yet. An earlier metabolomics study to characterize fecal samples from patients with CD and UC reported the results of analysis that include data showing a reduced level of trimethylamine, a precursor of TMAO [253]. A more recent study showed that plasma TMAO level was lower in the IBD compared to a non-IBD population, which was interpreted as a result of IBD-dependent loss of microbial diversity in the gut microbiome and it has been suggested as a potential biomarker for IBD [254]. Notably, this negative correlation of TMAO with IBD is contrary to the cases of cardiovascular and metabolic diseases [132].

As summarized in this section, over the past decades, roles of gut dysbiosis in the pathogenesis of IBD have been studied extensively in terms of changes in microbiome composition and the microbiota-derived metabolites. Particularly, recent multi-omics studies that characterized the functional dysbiosis in the gut microbiome through performing untargeted LC-MS metabolomic and shotgun metagenomics and metatranscriptomics profiling of stool samples from cohorts of CD, UC, and non-IBD control subjects, as part of the iHMP, has recently provided an improved understanding how disturbances of the gut microbiome-metabolome interface contribute to the pathogenesis of IBD [188,251]. The same research group has also created metagenomic, metatranscriptomic, and metabolomic profiles of host and microbial activity during IBD, providing a comprehensive description of IBD-associated gut dysbiosis [189].

## 6. Conclusions

Metabolic disorders, such as obesity, T2D, dyslipidemia, and non-alcoholic fatty liver disease are characterized by a dysregulation of energy metabolism and homeostasis, resulting in dysfunctional physiological consequences including dyslipidemia, glucose intolerance, oxidative stress, and chronic inflammation. IBD is a group of chronic inflammatory disorders in the gastrointestinal tract including the small intestine and colon, characterized by inflammation of the intestinal mucosa and abnormal responses of the innate and adaptive immune system. Despite considerable epidemiological evidence of comorbidities between these two pathophysiologically overlapping conditions, the molecular-level relationship in their pathogenesis has been poorly described so far. This review focused on the pathogenic correlation between them at molecular levels to explore the role of molecular links in each disease, which could provide opportunities for developing new therapeutic approaches based on targeting their common links. As summarized and discussed above, the pathogenesis of metabolic disorders has been found to be associated with metabolic-enteroendocrine dysfunction, intestinal barrier dysfunction, intestinal immune dysregulation, and specific alterations in the gut microbiota, all of which are shared as risk factors also in the development of IBD. This close mechanistic correlation between the pathogeneses of the two diseases shown in this review provides an integrative understanding of molecular mechanisms underlying their comorbidity.

Consequently, this advanced understanding can provide valuable insights into devising novel therapeutic approaches in the treatment of metabolic disorders and IBD. As an example, therapeutic strategies or agents to restore the pathogenic impairment of adipose tissue function, enteroendocrine function, intestinal barrier integrity, and intestinal immune function that connects the two diseases could be developed. Attention also should be paid to the fact that the characteristics of specific alterations in intestinal microbiota and their derived metabolites in each of the two diseases are mostly similar, excepting a few differences. In addition to dysbiosis itself, alterations in intestinal immunity induced by intestinal barrier dysfunction and dysbiosis should also be considered as a crucial link in understanding molecular mechanism underlying the comorbidity of metabolic disorders in IBD patients and vice versa. In this regard, probiotic therapy to modulate the specifically altered composition and function of the gut microbiota could be a strong strategy for the treatment of metabolic dysfunctions in patients with UC or CD. For example, a specific therapy based on anti-inflammatory effects of probiotics and their metabolites, a fecal microbiota transplantation, or a recently emerging gut health program including microbiome testing and personalized probiotics could be excellent treatment candidates. Given that metabolic disorders and IBD are diseases that require long-term treatment and minimizing the side effects is utmost critical in the process of treatment, the importance of probiotic therapy could be more emphasized compared to chemotherapy.

Finally, it should be stressed that although this review has focused on the representative pathophysiological features that appeared in both metabolic disorders and IBD, the connections between the two diseases are presumed to be more complicated since, in addition to the risk factors discussed in this review, others such as genetic and environmental risk factors, are also involved in their pathogeneses, suggesting that further studies are necessary to completely understand the mechanisms that explain the high comorbidity between them.

## Figures and Tables

**Table 1:** Molecular mechanisms underlying the impairment of adipose tissue function, enteroendocrine function, intestinal barrier integrity, and intestinal immune function in metabolic disorders and IBD.

Conditions	Metabolic Disorders	IBD
Adipose tissue dysfunction	↓adiponectin, ↓omentin-1, ↑leptin, ↑resistin, ↑visfatin, and ↑chemerin in obesity and/or T2D * [40,41,42]	↓adiponectin, ↓omentin-1, ↓or↑leptin in IBD * [45,46,47], ↑resistin, ↑visfatin, and ↑chemerin in IBD * [44,45,46,47]
Enteroendocrine cell dysfunction	↓GLP-1 [49], ↓GLP-2 [50,51], and ↑DPP-4 in obesity and T2D [56]	↑GLP-1 and ↑GLP-2 in IBD [58] ↓DPP-4 in IBD [60]
Intestinal barrier dysfunction	↓ZO-1, occludin in DIO and ob/ob mice * [50,65] ↓ZO-1, occludin, claudin-1 in rodent DIO models * [66] ↓claudin-1, -3, -4, -7, -15, and ↑claudin-2 (the leaky claudin) in rodent DIO models * [67] ↓occludin, tricellulin in obese patients with T2D * [68] ↓fecal zonulin in obese patients * [69] ↓ZO-1, occludin, but no change of claudin-1 in NAFLD patients* [70]	↓claudin-4 in both CD and UC * [71] ↓claudin-5, -8 in CD * [72] ↑claudin-1 in active forms of both CD and UC * [73,74,75], but no change in inactive forms [74] ↑claudin-2 in both CD and UC * [71,72,74], ↓occludin in CD * [72,75,76], no change [74]↓ZO-1 in colonic epithelial cell culture [76] and DSS-induced colitis mice * [75] Polymorphisms of genes encoding tight junction-associated proteins in IBD patients [77,78] ↑apoptosis [79,80,81,82], and ↑necrosis, necroptosis [79,83,84] in IBD patients
Intestinal immune dysfunction	Altered Th17/Treg balance in obesity, T2D, and NAFLD * [85] ↑pro-inflammatory cytokines in DIO mice [86,87] ↑IL-17-producing γδ T cells [87,88] and ↑IFN-γ-producing Th1 and CD8^+^ T cells [87] in DIO mice ↓IL-22 in db/db, ob/ob, and DIO mice [89] ↓Tregs in DIO mice * [85,86,87,89,90] ↓eosinophils in DIO mice [91]	Altered Th1/Th2 [92], Th1/Th17 [93,94], Th1/Treg [95] and/or Th17/Treg balance [92,94,95] in IBD * ↑Th1 responses (↑IFN-γ) in CD * [96,97] ↑Th2 responses (↑IL-4 and IL-5) in UC * [96,98] ↑Th17 (↑IL-17, IL-21, and IL-22) in IBD * [99] ↑Th1 or Th17 responses (↑IFN-γ and IL-17) in CD, and↑NK T (↑IL-13), IL-4, IL-5 in UC [98,100] ↑Th1-like Th17 (↑IFN-γ and IL-17), T17-like Treg in CD [101] ↑Th1-like Treg (↑IFN-γ) in both CD and UC [102] ↑plasticity of ILC3s (NKp44^+^NKp46^+^) to ILC1s (NKp44^−^NKp46^−^) and ↑plasticity of ILC2s to ILC1s in CD [103,104] ↓ILCreg in IBD [105,106]

* Possible links between metabolic disorders and IBD.

**Table 2:** Alterations in gut microbiota in metabolic disorders and IBD.

Metabolic Disorders	IBD
**In obesity** No difference in bacterial diversity [156] (phylum) ↑Firmicutes/Bacteroidetes [155,156], ↑Proteobacteria * [162] (class) ↓Clostridia * [162] (order) ↓Clostridiales * [162] (family) ↑Lachnospiraceae [160,162], ↑Bacilli, Enterobacteriaceae *, Lactobacillaceae, Streptococcaceae [162], ↓Christensenellaceae [158,162], ↓Clostridiaceae *, Dehalobacteriaceae, Ruminococcaceae * [162] (genus) ↑*Bacillus,* opportunistic pathogens (*Escherichia* *, Fusobacterium, Shigella *) [161], ↑ *Fusobacterium, Ruminococcus* * [160], ↑*Blautia* * [162], ↓*Bifidobacterium* *, Faecalibacterium * [161], ↓*Oscillospira* [158,162], (species) ↑*Lactobacillus reuteri* [157], ↑*Bacteroides fragilis, Lactobacillus* spp. [159], ↓*Bifidobacterium animalis* [157], ↓*Methanobrevibacter smithii* [157,158], ↓*Akkermansia muciniphila* [90], ↓*Bifidobacterium* spp. * [159] **In T2D** No difference in bacterial diversity [163] (order) ↑Clostridiales [164], ↓Clostridiales* [165] (genus) ↑*Lactobacillus* [164], ↑*Dorea, Ruminococcus* *, Sutterella and *Streptococcus* (in prediabetes) [165], ↑*Ruminococcus* * [165,166,167], ↑*Blautia* * [166,168], ↑*Fusobacterium* [169], ↑pro-inflammatory bacteria (*Escherichia* *, Enterobacter, Methanobrevibacter, Treponema) [170], ↓*Bacteroides* [166,167,168,171], ↓*Roseburia* * [164,166,167,172], ↓*Clostridium* [164,165], ↓*Bifidobacterium* * [167,169], ↓anti-inflammatory (*Anaerostipes, Blautia, Coprococcus, Lachnospira, Phascolarctobacterium, Roseburia* *) [170] (Species) ↑opportunistic pathogens (*Bacteroides caccae*, *Clostridium hathewayi*, *C. ramosum, C. symbiosum, Eggerthella lenta, E. coli* *) [163], ↑opportunistic pathogens (*Clostridium clostridioforme, C. bolteae, C. hathewayi, Streptococcus mutans*)*, Lactobacillus gasseri* [164], ↑genes belong to *Akkermansia muciniphila* [163], ↓*Faecalibacterium prausnitzii* * [163,164,166], ↓butyrate-producing bacteria (*Clostridiales* sp. SS3/4, *Eubacterium rectale, F. prausnitzii* *, Roseburia intestinalis *, Roseburia inulinivorans *) [163], ↓*Akkermansia muciniphila* [90,165,166,173] (in mesenteric adipose tissue) ↓Bacterial diversity, ↑Enterobacteriaceae *, ↓*Faecalibacterium* * [174](in plasma) ↑Enterobacteriaceae * [174] **In NAFLD** ↓Bacterial diversity [175] (phylum) ↑Proteobacteria * [176,177,178], ↑Verrucomicrobia [178](class) ↑Bacteroidia [179], ↓Clostridia * [179](family) ↑Enterobacteriaceae * [180], ↑Kiloniellaceae, Pasteurellaceae, Veillonellaceae * [176], ↓Rikenellaceae [175,180], ↓Bifidobacteriaceae [180], ↓Ruminococcaceae [176,179,180] (genus) ↑*Dorea* [175,176], ↑*Peptoniphilus* [175,180], ↑*Escherichia* * [178,180], ↑*Ruminococcus* * [175,181], ↑*Bacteroides* [181], ↑*Bradyrhizobium, Anaerococcus, Peptoniphilus* [175], ↑*Acidaminococcus, Akkermansia, Eggerthella, Flavonifractor* [178], ↓*Coprococcus* [178,179,180], ↓*Eubacterium* [178,180], ↓*Faecalibacterium* * [180], ↓*Prevotella* [178,181], ↓*Anaerosporobacter* [179], ↓*Oscillospira* [175] (species) ↑ *Clostridium coccoides, E. coli* * [180], ↑*Propionibacterium acnes* [175,181], ↓*Bacteroides fragilis* [181]	**In both CD and UC** ↓Bacterial diversity [182,183](phylum) ↓Bacteroidetes [182,184], ↓Firmicutes [182,184] class) ↓Clostridia * [182], ↑Gammaproteobacteria [182] (family) ↑Bacteroidaceae [185], ↓Ruminococcaceae * [182,185,186], ↓Clostridiaceae * [185](genus) ↑*Escherichia* *, (transcription levels of) *Blautia* * and *Lachnoclostridium* [187], ↓*Bacteroides, Bifidobacterium* *, Lactobacillus [182], ↓ *Alistipes, Faecalibacterium* *, Roseburia *, Subdoligranulum [187] (species) ↑*Bacteriodes fragilis, B. vulgatus* [185,188], ↑*E. coli* *, Klebsiella pneumonia [185,189], ↑*Alistipes putredinis, Roseburia intestinalis* * [188], ↑(mucolytic) *Ruminococcus gnavus* * [188,189,190,191], ↑*Ruminococcus torques* [189], ↑(transcription levels of) *Clostridium bolteae*, *Clostridium hathewayi*, and *Ruminococcus gnavus* * [189], ↓(butyrate-producing) *Faecalibacterium prausnitzii* *, Roseburia hominis * [15,182,185,189,192,193,194] **In CD** (phylum) ↑Proteobacteria * [195], ↓Bacteroidetes [196] (order) ↑Clostridiales [197], ↓Bacteroidales, Clostridiales *, Erysipelotrichales [198] (family) ↑Enterobacteriaceae * [182], ↑Fusobacteriaceae, Pasteurellacaea, Veillonellaceae * [198], ↑Ruminococcaceae [197] (genus) ↑*Escherichia* * [185,192], ↑*Enterococcus, Klebsiella, Veillonella* [192], ↑*Shigella* * [185], ↑*Faecalibacterium, Fusobacterium* [197], ↓ *Akkermansia, Alistipes, Coprococcus, Dorea, Eubacterium, Odoribacter, Parabacteroides, Prevotella, Roseburia* *, Ruminococcus [192] (species) ↓*Bifidobacterium longum* [185], ↓*Faecalibacterium prausnitzii* *, Roseburia hominis * [189] **In UC** (phylum) ↑Actinobacteria, Proteobacteria * [199], ↓Bacteroidetes, Verrucomicrobia [199] (order) ↓butyrate-producers of Clostridiales * [200] (family) ↑Enterobacteriaceae * [198], ↑Staphylococcaceae, Streptococcaceae, Veillonellaceae [199], ↓Akkermansiaceae, Bacteroidaceae [199] (genus) ↑*Bacillus, Escherichia* *, Shigella *, Peptostreptococcus, Veillonella [199], ↓*Akkermansia, Bifidobacterium* *, Faecalibacterium * [199] (species) ↓*Faecalibacterium prausnitzii* *, Roseburia hominis * [194]

* Possible links between metabolic disorders and IBD.

**Table 3:** Alterations in gut microbiota-derived metabolites in metabolic disorders and IBD.

Metabolic Disorders	IBD
**SCFAs**↓(plasma) acetate in hyperinsulinaemic humans [218], ↓(plasma) total SCFAs, and ↓SCFA producing bacteria in high-fat/high-sucrose-diet fed mice [219] ↓(fecal) acetate and butyrate *, and ↓SCFA producing bacteria * in T2D patients [222] ↓(cecal) acetate, butyrate, and propionate * (↓*Akkermansia muciniphila*) in aged mice with insulin resistance [228] ↓Lachnospiraceae, and ↓NLRP12 in DIO mice [226] ↓propionate-induced GLP-1 and PYY in overweight adults [220,224] ↓bacterial genes for butyrate and propionate production * in T2D patients [172] ↓acetate- and butyrate-induced GLP-1 and PYY in T2D patients [222] Restored gut dysbiosis and intestinal barrier dysfunction (↑ZO-1 [221] and ↑GLP-1R [223]) in butyrate-treated HFD-induced NAFLD mice ↑(cecal, fecal) SCFAs in obese mice and human subjects [228] **BAs** ↑(plasma) total BAs * and total glycine conjugated BAs in obese patients with T2D [229] ↓(fecal) BSH gene * in T2D patients [230] ↑(plasma) primary BAs *, taurocholate, and total conjugated cholate, and ↓total secondary/primary BAs * in NAFLD patients [231] ↓ total secondary/primary BAs *, and ↓(plasma) unconjugated BAs in T2D patients treated with acarbose [232] ↓(fecal) cholate, deoxycholate, and total BAs content in obese patients supplemented with dietary fiber [233] ↓ (fecal) taurocholic acid in obese patients transplanted with fecal microbiota from lean donor [234] **TMAO** ↑(plasma) TMAO and ↓phosphatidylcholine in T2D mice [235] ↑(plasma) TMAO in T2D patients [236,237] ↑(plasma) TMAO and FMO3 (TMAO-producing enzyme) in T2D patients and DIO mice [238] Higher BMI in individuals in the highest plasma TMAO category [239] **Tryptophan and indole-derivative metabolites** ↑(plasma) kynurenine/tryptophan ratio (IDO1 activity) * and (adipose tissues and liver) IDO1 expression in obese patients [240] ↑(plasma) kynurenine * and (adipose tissues) kynurenine pathway enzymes in obese patients [241] ↑(plasma, fecal) kynurenine and IDO1 activity *, and ↓(fecal) indole-3-acetic acid (IAA) * in obesity and T2D [115] ↑(fecal) kynurenine * and ↓(fecal) AHR agonists (IAA, indole, 3-methylindole, and tryptamine) * in patients with metabolic syndrome [242] ↑(intestine) GLP-1 and IL-22 in AHR agonist-treated DIO mice [242] ↓(plasma) indole and↑(WAT) miR-181 family in obese mice and humans [243] **BCAAs** ↑insulin resistance in BCAA-supplemented HF-fed rats [244] ↑(plasma) BCAA in T2D patients [245,246] ↑(plasma) branched-chain keto-acid (BCKA, converted from BCAAs) in T2D patients [246] ↑(plasma) 3-hydroxyisobutyrate (a valine metabolite) in db/db mice and T2D patients [247] ↑(gut microbiome) BCAA biosynthetic genes in T2D patients [202] ↑(plasma) BCAA and BCKA, and ↓insulin sensitivity by BCAA supplementation in *ob/ob* mice [248]	**SCFAs** ↓butyrate-synthetic capacity * in patients with active and inactive CD, and with active UC [249] ↓(fecal) butyrate * (↓Clostridiales) and ↑NLRP1 inflammasome, IL-18, and IFN-γ in DSS-colitis mice [200] ↓an anti-inflammatory protein from butyrate-producing *Faecalibacterium prausnitzii* in colitis mice [15] ↑(serum, fecal) acetate, ↑GPR43 and GPR109A activation, and ↑NLRP3 inflammasome activation by high-fiber feeding in DSS-colitis mice [121] **BAs** ↓(fecal) bile salt biotransformation gene abundance *, and ↑primary BAs * (cholate, chenodeoxycholate, glycocholate, taurocholate, taurochenodeoxycholate), and ↓secondary BAs * (deoxycholate, lithocholate) in IBD patients [250,251] ↓(fecal) BSH gene * in UC patients [230] ↑(gallbladder) taurocholate in high saturated-fat diet-fed Il10^-/-^ mice [252] **TMAO** ↓(fecal) trimethylamine [253] ↓(plasma) TMAO in IBD patients [254] **Tryptophan and indole-derivative metabolites**↓(plasma) tryptophan *, ↑(plasma) quinolinic acid * in IBD patients [255] ↓(intestine) AHR * in IBD patients [256] ↓IFN-γ and ↑IL-22 in intestinal lamina propria mononuclear cells of IBD patients treated with an AHR agonist [256] ↓(fecal) tryptophan, AHR activity, and IAA *, and ↑kynurenine * [257], and an association with single-nucleotide polymorphism within *CARD9* gene in IBD patients and germ-free mice colonized with the microbiota of CARD9-deficient mice [257] ↓(colon, serum) indole, indole derivatives, and indole-3-propionic acid (IPA) * in DSS-induced colitis mice [258] ↓(serum) IPA * in UC patients [258] ↑IL-10R1 in germ-free mice colonized with *E. coli* mutants unable to generate indole [258]↓(fecal) the genetic capability of microbes (*Peptostreptococcus* spp.) to metabolize tryptophan into indoleacrylic acid in IBD patients [259] **BCAAs** ↓(plasma) BCAA leucine and valine (correlated with disease activity) in CD patients [260] No correlation of plasma BCAAs with UC, but negatively correlated with some UC-related parameters such as calprotectin and age [261]

* Possible links between metabolic disorders and IBD.

## Data Availability

Not applicable.

## Abbreviations

AIM2	Absent in melanoma 2
AMP	Antimicrobial peptide
AHR	Aryl hydrocarbon receptor
BA	Bile acid
BSH	Bile salt hydrolase
BMI	Body mass index
BCAA	Branched-chain amino acid
CARD9	Caspase recruitment domain family member 9
CCR	CC chemokine receptor
CCL20	Chemokine ligand 20
CD	Crohn’s disease
DIO	Diet-induced obesity
DPP4	Dipeptidyl peptidase-4
FXR	Farnesoid X receptor
GLP-1R	GLP-1 receptor
GLP-1	Glucagon-like peptide-1
GLP-2	Glucagon-like peptide-2
GPR	G-protein-coupled receptor
HFD	High fat diet
IPA	Indole-3-propionic acid
IDO1	Indoleamine 2,3-dioxygenase 1
IBD	Inflammatory bowel disease
ILC	Innate lymphoid cell
IFN-γ	Interferon-gamma
IEC	Intestinal epithelial cells
LPS	Lipopolysaccharide
MAMP	Microbial-associated molecular pattern
NCR	Natural cytotoxicity receptor
NLR	NOD-like receptors
NAFLD	Non-alcoholic fatty liver disease
NASH	Non-alcoholic steatohepatitis
NOD	Nucleotide-binding oligomerization domain
PAMP	Pathogen-associated molecular pattern
PRR	Pattern recognition receptor
Treg	Regulatory T cell
sIgA	Secretory immunoglobulin A
SCFA	Short-chain fatty acid
Tfh	T follicular helper
Th	T helper
TLR	Toll-like receptor
TAK	Transforming growth factor-activated kinase
TMAO	Trimethylamine N-oxide
TNF-α	Tumor necrosis factor-alpha
T1D	Type 1 diabetes
T2D	Type 2 diabetes
UC	Ulcerative colitis
ZO-1	Zonula occluden-1
